# Enhanced Cycling Stability of Cation Disordered Rock-Salt Li_1.2_Ti_0.4_Mn_0.4_O_2_ Material by Surface Modification With Al_2_O_3_

**DOI:** 10.3389/fchem.2019.00107

**Published:** 2019-03-04

**Authors:** Baojun Huang, Rui Wang, Yansheng Gong, Beibei He, Huanwen Wang

**Affiliations:** Faculty of Materials Science and Chemistry, China University of Geosciences, Wuhan, China

**Keywords:** lithium-ion batteries, cathode, cation disorder, rock-salt, Li-excess

## Abstract

Cation disordered rock-salt lithium-excess oxides are promising candidate cathode materials for next-generation electric vehicles due to their extra high capacities. However, one major issue for these materials is the distinct decline of discharge capacities during charge/discharge cycles. In this study, Al_2_O_3_ layers were coated on cation disordered Li_1.2_Ti_0.4_Mn_0.4_O_2_ (LTMO) using atomic layer deposition (ALD) method to optimize its electrochemical performance. The discharge capacity after 15 cycles increased from 228.1 to 266.7 mAh g^−1^ for LTMO after coated with Al_2_O_3_ for 24 ALD cycles, and the corresponding capacity retention enhanced from 79.7 to 90.9%. The improved cycling stability of the coated sample was ascribed to the alleviation of oxygen release and the inhibition on the undesirable side reactions. Our work has provided a new possible solution to address some of the capacity fading issues related to the cation disordered rock-salt cathode materials.

## Introduction

Cathode materials with high energy densities are crucial for next generation of lithium-ion batteries, especially when used in hybrid and electric vehicles (Zu and Li, 2011; Goodenough and Park, 2013; Rui et al., 2015; Wang et al., 2017; Lv et al., 2018; Tan et al., 2018; Zhang et al., 2019). For this reason, Layer-structured Li-excess materials, which could deliver capacities as high as 300 mAh g^−1^, have been researched for more than 10 years (Lu et al., 2002; Wang et al., 2013; Yu et al., 2014; Hu et al., 2018). But these materials undergo irreversible O loss in the first cycle, which may cause structure densification and voltage degradation in subsequent cycles (Xu et al., 2011). Recently, cation disordered rock-salt Li-excess materials, sharing similar chemical compositions with layered Li-excess materials, have also attracted lots of attentions because of their high capacities (Yabuuchi et al., 2011, 2015, 2016a,b; Lee et al., 2014, 2015, 2017, 2018; Twu et al., 2015; Freire et al., 2016, 2017; Cambaz et al., 2018; Kitchaev et al., 2018; Zhao et al., 2019). Besides, it is reported that the O redox may undergo reversible reactions in the first cycle in this material (Wang et al., 2015), which provides the possibility to achieve reversible changes during the lengthy electrochemical cycles. However, many of these materials have problems in their cycling performances. Wang et al. prepared a cation disordered Li_1.23_Ni_0.155_Ru_0.615_O_2_ material, and the capacity drops from 295.3 to 250 mAh g^−1^ only after 5 charge-discharge cycles (Wang et al., 2017). Okada's group prepared another cation disordered Li_1.2_Mn_0.4_Ti_0.4_O_2_ material, but its capacity drops from 226 to about 200 mAh g^−1^ just after 6 cycles (Kitajou et al., 2016).

Similar problems happened to layered Li-excess materials, and some literatures have reported that atomic layer deposition (ALD) method may be an effective method to alleviate the problem. ALD is a powerful technique to precisely render a uniform and conformal layer at Å level on arbitrary substrate surfaces due to its pulsing and controllable reaction. Belharouak's team coated Li_1.2_Ni_0.13_Mn_0.54_Co_0.13_O_2_ porous powder with ultrathin Al_2_O_3_ film using ALD technique, and the coated material shows higher first cycle coulombic efficiency and improved cycling performance (Zhang et al., 2013). Xiao et al. reported that the AlPO_4_ coating layer by ALD can effectively protect the Li_1.2_Mn_0.54_Co_0.13_Ni_0.13_O_2_ against the attack from the electrolyte, and can significantly improve its initial coulombic efficiency and thermal stability (Xiao et al., 2017a). Meanwhile, Oxide-based coatings at the surface of different cathode materials via ALD method have been demonstrated to be very conformal and uniform as reported in the literatures (Zhao and Wang, 2012; Zhao et al., 2012; Zhao and Wang, 2013a,b), thus they can effectively prevent from the electrolyte attack for enhanced cycling stability of the coated cathode.

In this study, we coated a cation disordered rock-salt Li_1.2_Ti_0.4_Mn_0.4_O_2_ material with different thicknesses of Al_2_O_3_ layers by ALD technique and studied their effects on the cycling stabilities. Li_1.2_Ti_0.4_Mn_0.4_O_2_ is a typical cation disordered rock-salt material but with poor cycling performances (Kitajou et al., 2016; Yabuuchi et al., 2016a). In our previous study, we found that the valence of Mn in disordered materials may be lower than 3+ after the first cycle (Wang et al., 2015), and this low valence may cause Mn dissolution into the electrolyte (Nicolau et al., 2018). In this case, we infer that Al_2_O_3_ coating may be an effective method to increase the reversibility and cycling performances of this cation disordered material.

## Experimental

Li_1.2_Ti_0.4_Mn_0.4_O_2_ was synthesized by the traditional solid-state reaction using the precursors of Li_2_CO_3_ (99%, Alfa Aesar), Mn_2_O_3_ (98%, Alfa Aesar), and TiO_2_ (99%, Sigma-Aldrich). Stoichiometric amounts of precursors were ball-milled for 4 h and then pressed into a pellet. The pellet was calcinated at 900°C for 12 h in Ar, then pulverized and ball-milled at 300 rpm for 4 h. The obtained material was denoted as LTMO.

Coating was achieved by the Atomic Layer deposition (ALD) method using an Ensure Nanotech ALD system (LabNano-9100). During the ALD process, as-prepared LTMO sample was first placed in a home-made sample holder and heated to 200°C in the reaction chamber under ca. 1.0 mbar. Nitrogen with a flow rate of 20 sccm was used as the carrying and purge gas. The Al(CH_3_)_3_ and water were employed as aluminum and oxygen precursors, respectively. In each cycle, pulse time of Al(CH_3_)_3_ was controlled at 0.02 s, and exposure time water precursor was 0.02 s. 8 s nitrogen purge steps were used after Al(CH_3_)_3_ and water exposures. The LTMO sample was coated with Al_2_O_3_ layers for 16, 24, 40 ALD cycles. The coated samples were denoted as LTMO/nAl_2_O_3_, where n stands for the number of ALD cycles.

Morphologies and crystal structures of LTMO with or without Al_2_O_3_ layers were analyzed by scanning electron microscope (SEM, SU 8010) and powder X-ray diffractometer (XRD, Bruker D8 Advance), respectively. High Resolution Transmission Electron Microscope (HRTEM, TF 20) was used to record detailed crystalline structures of the samples. X-ray photoelectron spectroscopy (XPS, ESCALab 250Xi) with monochromatic Al K-α radiation was carried out to investigate the valences of the species in the samples.

To fabricate the cathode electrode, different Al_2_O_3_-coated LTMO samples were firstly ball-milled with Ketjen black at 150 rpm for 4 h, then manually mixed with polytetrafluoroethylene (PTFE) binder. The mixture was rolled into a thin film, in which the weight ratios of LTMO/nAl_2_O_3_, Ketjen black, and PTFE are 70: 20: 10. The surface mass density of each electrode film is about 4.4 mg cm^−2^. Cells were assembled according to our previous study (Wang et al., 2015). Typically, a two electrode swagelok cell was fabricated using the Al_2_O_3_-coated LTMO thin film and lithium foil as the working electrode and the counter electrode, respectively. Borosilicate glass fiber membrane (Whatman) was used as the separator, and 1 M solution of LiPF_6_ dissolved in ethylene carbonate/dimethyl carbonate (1:1 by volume) was used as the electrolyte. All cells were cycled on an LANHE CT2001A system (Wuhan LAND Electronics Co.) between 4.8 and 1.5 V with a current density of 10 mA g^−1^ at room temperature. The electrochemical impedance spectroscopy (EIS) data were recorded using a Gamry Reference 3,000 equipment. Cyclic voltammetry (CV) was measured on a CHI760E electrochemical workstation with the scanning rate of 0.5 mV s^−1^ and the potential range of 4.8–1.5 V.

## Results and Discussion

**Figure 1:**
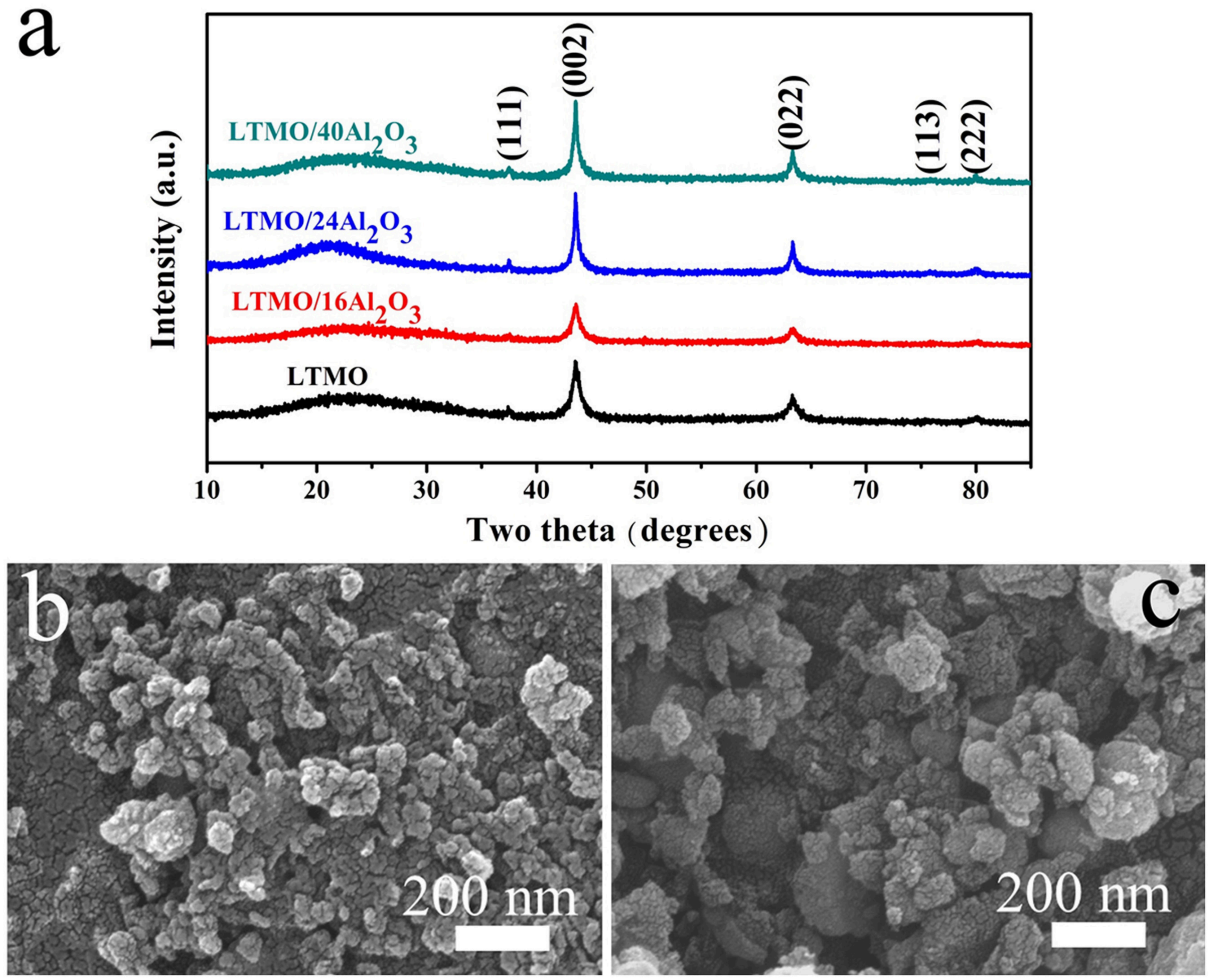
**(a)** XRD pattern of the bare LTMO and different Al_2_O_3_-coated LTMO; SEM image of **(b)** LTMO and **(c)** LTMO/24Al_2_O_3_.

**Figure 2:**
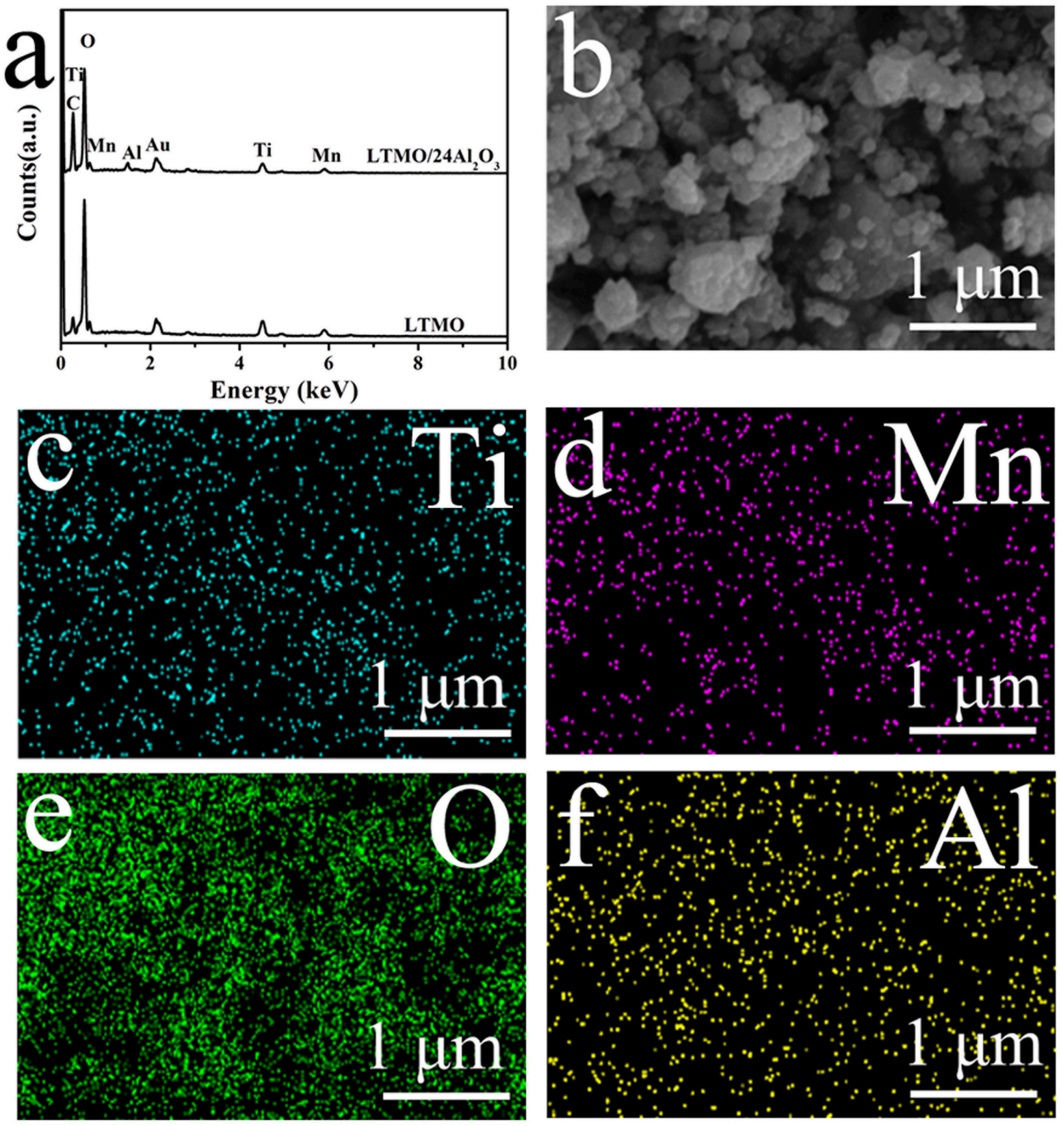
**(a)** EDS element analysis LTMO and LTMO/24Al_2_O_3_; **(b–f)** element mapping of LTMO/24Al_2_O_3_.

X-ray diffraction (XRD) patterns of the samples are shown in Figure 1a, and all the results correspond well to the disordered rock-salt structure. According to previous studies, cations are randomly placed on the 4b position (1/2, 1/2, 1/2), and the O atom is placed on the 4a position (0, 0, 0) (Wang et al., 2015). In cation disordered rock-salt materials, Li excess could build the 0-TM channels, through which Li ions could move out and back into the cathode material (Lee et al., 2015). According to the XRD patterns, no signal of Al_2_O_3_ is detected, and the reason may be that the amount of coated Al_2_O_3_ is too small or the coated Al_2_O_3_ is amorphous. The morphologies of LTMO before and after Al_2_O_3_ coating are shown in Figures 1b,c. No obvious differences are observed, indicating the coated Al_2_O_3_ particle may be too small to be surveyed in SEM. Energy Dispersive Spectrometers of the two samples are shown in Figure 2a. The characteristic peak of Al element could clearly be observed in the LTMO/24Al_2_O_3_ sample, while didn't show up in the LTMO sample. The LTMO/24Al_2_O_3_ sample was further examined by the elemental mapping experiments. and the results are shown in Figures 2b-f. Results show that Ti, Mn, O and Al elements distribute uniformly in the sample, which infer the Al_2_O_3_ layer exists uniformly in the sample.

**Figure 3:**
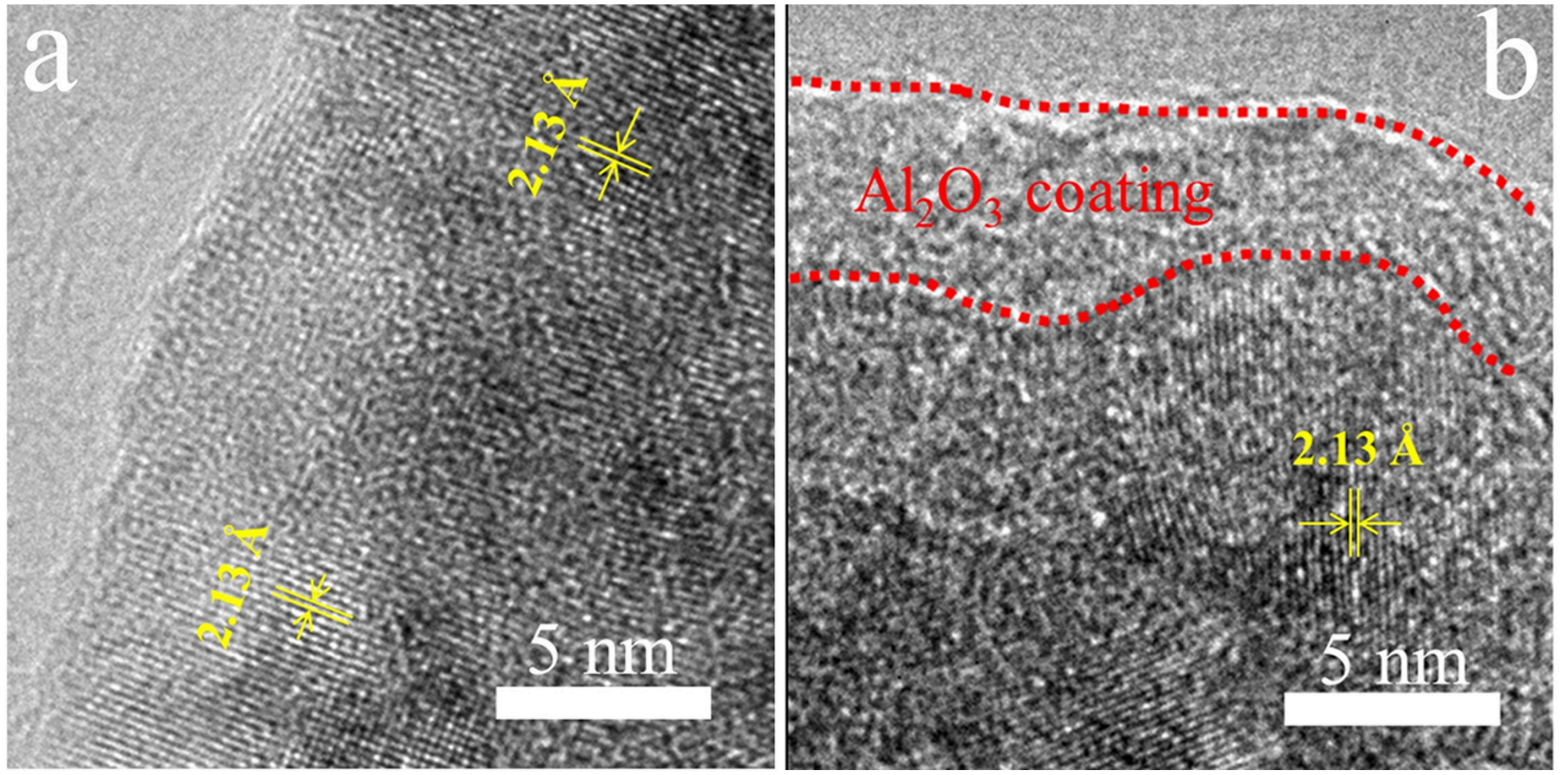
HR-TEM images of **(a)** LTMO and **(b)** LTMO/24Al_2_O_3_.

In order to directly observe the coating Al_2_O_3_ layers on the surface of LTMO particles, we investigate bare LTMO and LTMO/24Al_2_O_3_ samples using high resolution transmission electron microscopy (HR-TEM). Figure 3a is the HR-TEM image of bare LTMO particles, showing well-defined lattice fringes in the surface region as well as those in the bulk region. In contrast, an obvious coating film in the surface region is clearly observed for LTMO/24Al_2_O_3_ sample (Figure 3b). It is different from the inner areas, and we infer it may be the Al_2_O_3_ coating layer. The thickness of the coating layer is around 3–5 nm.

**Figure 4:**
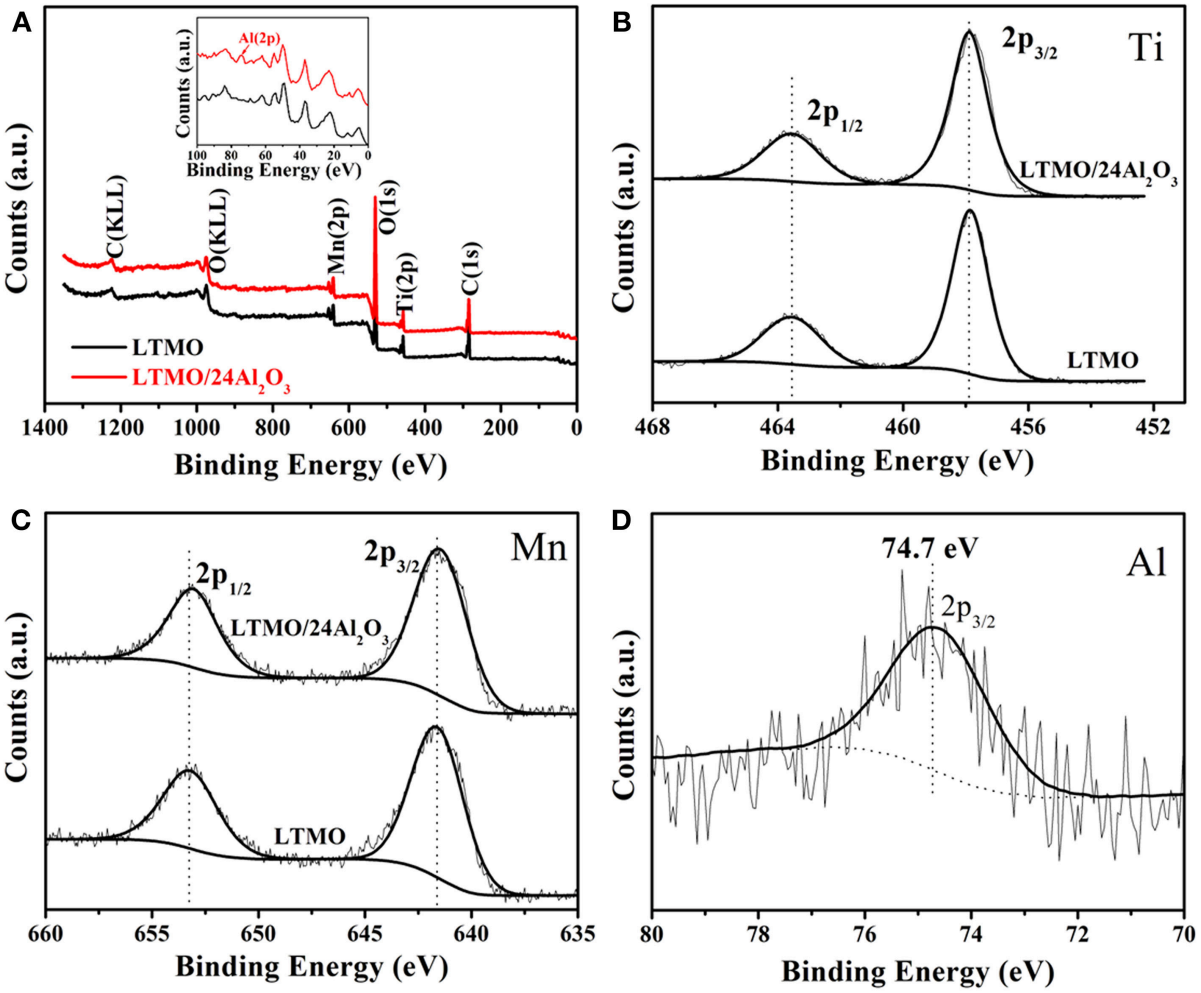
**(A)** XPS survey spectrum and core level spectra of transition metals of LTMO and LTMO/24Al_2_O_3_: **(B)** Ti 2p; **(C)** Mn 2p; **(D)** Al 2p.

In order to confirm Al_2_O_3_ layer exists on the sample surfaces, XPS was used to probe the surface compositions of the LTMO and LTMO/24Al_2_O_3_. All spectra were calibrated with the C 1s peak at 284.6 eV. Figure 4A presents the elemental XPS spectra of LTMO and LTMO/24Al_2_O_3_ samples, showing both samples contain the characteristic peaks of Ti, Mn, O, and C. From the partial enlarged figure (inset of the Figure 4A), the elemental peak of Al is only observed in the LTMO/24Al_2_O_3_ spectrum. The core level binding energies of Ti are aligned at 457.9 eV (2p_3/2_) and 463.6 eV (2p_1/2_) (Figure 4B), which is in good agreement with Ti^4+^ ions in other titanium-based compounds (Kim et al., 2017). The Mn 2p XPS spectra of LTMO and LTMO/24Al_2_O_3_ reveal peaks at 641.6 eV and 653.2 eV (Figure 4C), which are characteristic peaks of Mn^3+^ 2p_3/2_ and 2p_1/2_ (Das et al., 2011, 2013). This result confirms the valences of Mn in both samples are 3+. The Al 2p_3/2_ core level XPS spectrum displays a binding energy of 74.7 eV for the LTMO/24Al_2_O_3_ (Figure 4D), affirming that the chemical composition of the surface coating is Al_2_O_3_ for the LTMO/24Al_2_O_3_ sample.

**Figure 5:**
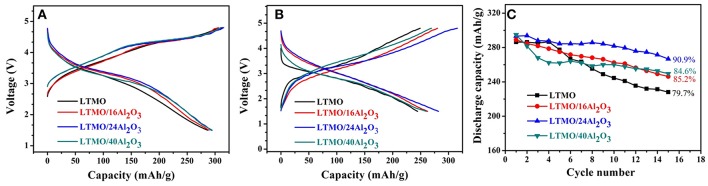
**(A)** Initial charge-discharge curves; **(B)** 10th charge-discharge curves; **(C)** cyclic performance of the pristine LTMO and Al_2_O_3_-coated LTMO electrodes.

The charge and discharge profiles of uncoated and coated LTMO samples for the first cycle are shown in Figure 5A, showing all the samples have the identical profiles except for the voltage platform and capacity. It is obviously seen that all the coated LTMO samples have higher first discharge plateau and coulombic efficiency, and we think this may be the effect of Al_2_O_3_ layer. Though the O reaction in LTMO may happen in the form of O redox, there might still be little amount of oxygen release in the first cycle. We infer the Al_2_O_3_ layer may alleviate this oxygen release and improve the O reaction reversibility, thus improve the discharge plateau and coulombic efficiency. The charge and discharge profiles for the 10th cycle are shown in Figure 5B, and the LTMO/24Al_2_O_3_ sample presents the largest discharge capacity. Figure 5C shows the cycling stability of the pristine and ALD coated samples. Bare LTMO shows an initial discharge capacity of 286.3 mAh g^−1^ (818.6 Wh kg^−1^) and the capacity drops rapidly to 228.1 mAh g^−1^ (587.7 Wh kg^−1^) after 15 cycles, which is only 79.7% for its initial discharge capacity. After coated with Al_2_O_3_ for 16 ALD cycles, an initial discharge of 289.1 mAh g^−1^ (856.6 Wh kg^−1^) is observed and the capacity retention reaches 85.2%. It is worth noting that the LTMO/24Al_2_O_3_ sample could deliver a capacity of 293.4 mAh g^−1^ and energy density of 884.5 Wh kg^−1^ in the first cycle. After 15 cycles, the capacity and energy density could still maintain 266.7 mAh g^−1^ and 709.5 Wh kg^−1^, respectively. And the capacity retention after 15 cycles is 90.9%, which is 11.2% higher than that of the pristine LTMO. Besides, the coulombic efficiencies also increased from 91.5 to 93.0% after the 24 cycles ALD coating. However, further increasing the ALD cycle numbers of coated Al_2_O_3_, the inferior performance for LTMO/40Al_2_O_3_ is obtained, and the capacity retention decreases to 84.6%. The reason should be the thick insulated Al_2_O_3_ layer may inhibit the electric and ionic transportation (Jung et al., 2010). Based on above results, it can be concluded that proper thickness of Al_2_O_3_ layer can greatly improve the cycling stability of LTMO electrodes, while the over-thick Al_2_O_3_ layer leads to the reverse effect on the electrode.

**Figure 6:**
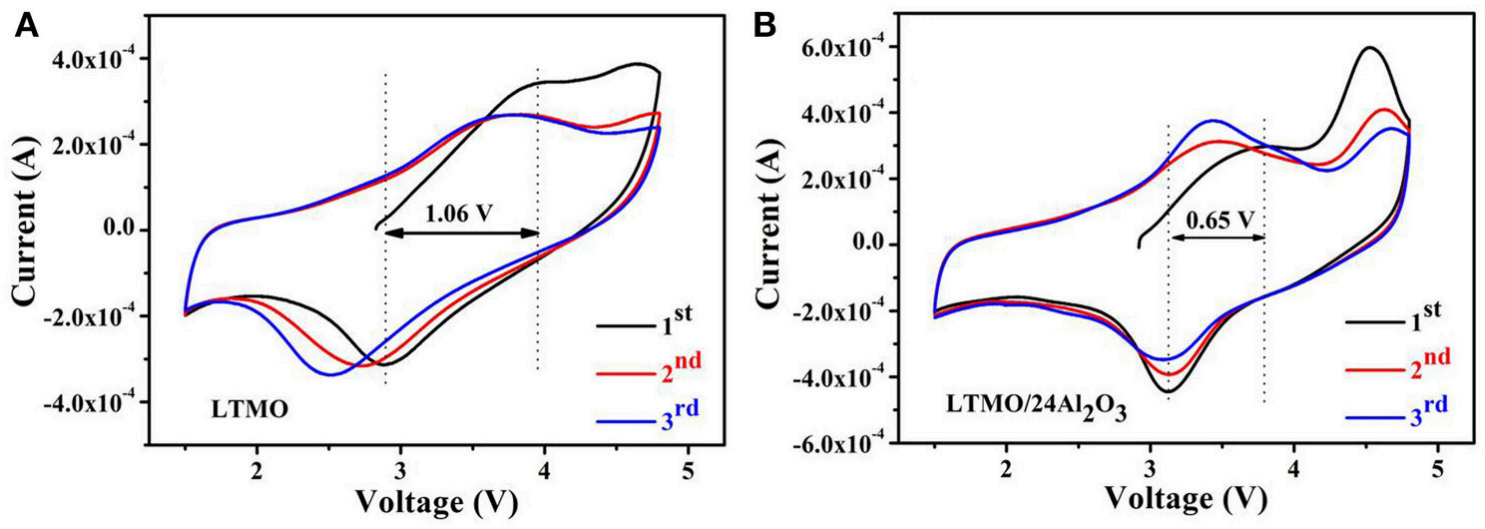
Cyclic voltammetry of **(A)** LTMO and **(B)** LTMO/24Al_2_O_3_.

Figure 6 show the cyclic voltammetric (CV) curves of LTMO and LTMO/24Al_2_O_3_ samples in the first three cycles at a scanning rate of 0.5 mV s^−1^. For the LTMO electrode (shown in the Figure 6A), the anodic peaks located at about 3.95 V corresponds to the oxidation of Mn^3+^ to Mn^4+^ (Xiao et al., 2017b) during the initial charge process. Another anodic peaks at around 4.63 V may be attributed to the oxygen loss from the crystal structure and formation of O${}_{2}^{2-}$ or O_2_ from O^2−^, which is usually observed on the Mn-based Li-excess cathode materials (Ma et al., 2016). While this peak disappears in the subsequent cycles, showing the irreversibility of the oxidation of O^2−^ in the LTMO electrode. In the following cathodic process, the peak at around 2.89 V corresponds to the reduction of Mn^4+^ to Mn^3+^ (Kong et al., 2017). It can be clearly seen that this peak moves to lower voltage range in the following cycles, indicating the decreasing of the valence of Mn. As to LTMO/24Al_2_O_3_ (shown in Figure 6B), two differences can be observed. First, the anodic peak at around 4.63 V still can be observed in the second and third cycles. Second, the cathodic peak located at around 3.17 V doesn't move during the cycles. These results infer the oxygen loss from the crystal structure in LTMO/24Al_2_O_3_ is milder than in LTMO. As a result, the Mn redox reaction in LTMO/24Al_2_O_3_ happens between Mn4+ and Mn3+. While Mn in pristine LTMO reduces to lower than 3+ because of the O loss and related densification in the first cycle.

**Figure 7:**
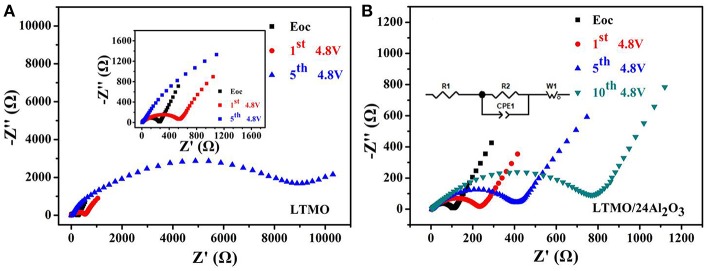
EIS profiles of **(A)** LTMO and **(B)** LTMO/24Al_2_O_3_ at the charge state of 4.8 V in the different cycles; inset: an equivalent-circuit simulation model.

In order to investigate the intrinsic factor of the improvement in the electrochemical performance of the LTMO/24Al_2_O_3_ sample, electrochemical impedance spectra (EIS) were collected on the bare LTMO and the LTMO/24Al_2_O_3_ after charging to 4.8 V and resting for 4 h at the different cycles, shown in Figures 7A,B, respectively. All the Nyquist plots comprised a depressed semicircle from high to middle frequencies and an inclined line at low frequency. The simulated equivalent circuit is presented as an inset. The R_1_ represents the Ohmic resistance coming from the separator, electrolyte and other components. The semicircle shows the charge transfer reaction composed of a charge transfer resistor (R_2_) and a constant phase element (CPE_1_), the inclined line stands for the Warburg diffusion impedance (ZW). The LTMO/24Al_2_O_3_ electrode shows the smaller R_2_ of 101.6 Ω before cycling and remains at 228.4 Ω, 433.8 Ω and 851.3 Ω after 1st, 5, and 10th cycling, respectively. Nevertheless, the much larger R_2_ values of the LTMO electrode are seen (i.e., 255.8 Ω before cycling and changed to 557.7 Ω after 1st cycling, even to 10,207 Ω for the 5th cycling, respectively), which infer complex side reactions may happen on the electrode surface and these reaction products may have blocked the ionic transfer process and result in worse cycling performance. Thus, it can be concluded that an appropriate thickness of Al_2_O_3_ layer guarantees the stable charge transfer and structural integrity of the cathode electrodes, which led to good cycling performance.

## Conclusion

ALD technique was successfully used to deposit ultrathin Al_2_O_3_ coating layer onto the surface of the LTMO particles. Comparing with the uncoated LTMO, ALD process can reduce the polarization, restrain the undesirable side reactions, and suppress the increasing charge transfer resistance during cycling, which results in the significantly improved electrochemical performance of Al_2_O_3_-coated LTMO (LTMO/24Al_2_O_3_). The controllable ALD technology provides a promising guideline for the surface modification of disordered rock-salt cathode materials with high electrochemical performance.

## Author Contributions

RW conceived and designed the research. BaH carried out the experiments. YG and HW contributed to the discussion. RW wrote the manuscript with the help of BeH. All authors reviewed the manuscript.

### Conflict of Interest Statement

The authors declare that the research was conducted in the absence of any commercial or financial relationships that could be construed as a potential conflict of interest.
